# Bioaugmentation of Entomopathogenic Fungi for Sustainable *Agriotes* Larvae (Wireworms) Management in Maize

**DOI:** 10.3389/fpls.2020.535005

**Published:** 2020-09-17

**Authors:** Jaka Razinger, Eva Praprotnik, Hans-Josef Schroers

**Affiliations:** Plant Protection Department, Agricultural Institute of Slovenia, Ljubljana, Slovenia

**Keywords:** biological control, biopesticide, plant–microbe interaction, plant–microbe–insect interaction, rhizosphere, sustainable agriculture, plant-microbe-pest interaction, biocontrol

## Abstract

Soil microorganisms influence biotic and abiotic stress tolerance of crops. Most interactions between plant symbiotic and non-symbiotic soil microorganisms and plants occur in the rhizosphere and are sustained through plant exudation/rhizodeposition. Bioaugmentation, *i.e.*, the introduction or amplification of certain plant beneficial microbes (*e.g.*, entomopathogenic fungi) into the rhizosphere, could contribute to controlling insect crop pests and replacing chemical, environmentally unfriendly insecticides. Wireworms, the soil-burrowing larval stages of click beetles (Coleoptera: Elateridae), are major pests of crops including maize, wheat and potatoes, worldwide. Alternative strategies for controlling wireworms are needed because several chemical pesticides used successfully in the past are being phased out because of their ecotoxicity. Therefore, virulence to *Agriotes* *lineatus* L. wireworms and plant beneficial traits of entomopathogenic fungi were investigated in a series of laboratory experiments. Tested taxa included environmentally retrieved *Metarhizium brunneum* Petch. (two strains), *M. robertsii* Bisch., Rehner & Humber (Hypocreales: Clavicipitaceae), and *Beauveria brongniartii* (Sacc.) Petch. and commercially formulated *B. bassiana* (Bals.-Criv.) Vuill. (Cordycipitaceae) and *Bacillus* *thuringiensis* Berliner 1915 var. *kurstaki*. In-house reared larvae were dipped in conidial suspension, and maize and wheat seeds were coated with fungal conidia. *Metarhizium brunneum* strains 1154 and 1868 significantly increased wireworm mortality. Fungi were significantly more often re-isolated from maize than wheat rhizoplanes in laboratory assays. The strains tested were rarely isolated as endophytes. *Metarhizium brunneum* strain 1154 stimulated wheat growth, while *M. robertsii* 1880 stimulated maize growth, whereas *M.* *brunneum* 1868 and others did not affect root or shoot length or plant biomass significantly in laboratory settings. *Metarhizium* *brunneum* strain 1868, re-isolated most often from maize rhizoplane, caused the highest wireworm mortality. It was further evaluated whether *M.* *brunneum* 1868 can protect maize varieties FeroXXY, LG 34.90 and Chapalu from wireworm damage and promote plant growth at field conditions. Plants of all three varieties stemming from seeds treated with conidia of *M. brunneum* 1868 showed significantly less wireworm damage 3 to 4 weeks after sowing (5- to 6-leaf stage) resulting in a significantly higher initial maize stand. However, only in the variety LG 34.90 a significant increase of the maize stand was observed at harvest time.

## Introduction

Wireworms (Coleoptera: Elateridae) damage potato and other crops including wheat and maize. They start feeding on seed potatoes shortly after planting without causing plant losses initially. However they reduce the market quality of the produce as they penetrate into near harvest potatoes (Benjamin et al., 2018). Once potatoes are damaged, also secondary microbial infections occur and the yield of entire potato crops can become unmarketable in high pest pressure areas or organic production settings (Brandl et al., 2017). Due to their hidden life cycle belowground, wireworms can hardly be controlled, especially in organic farming, where persistent, non-specific soil insecticides cannot be used (Schepl and Paffrath, 2007; Brandl et al., 2017; Benjamin et al., 2018). In maize and wheat, wireworms target germinating seeds and young sprouts, what results in typical herbivory symptoms such as leaf drilling holes and dead central leaves. However, in case of severe infestations, plant stand and yield can be significantly decreased (Reddy et al., 2014; Furlan et al., 2017). It has been emphasized that the abandonment of ecotoxicologically problematic soil insecticides may increase wireworm-related problems (Parker and Howard, 2001; van Herk and Vernon, 2013).

Organophosphates, organochlorines, and carbamates effectively controlled wireworms in the second half of the 20^th^ century (Merrill, 1952). However, due to their ecotoxicity (Costa, 2015), biomagnification in non-target organisms (Mitra et al., 2011), and yearlong availability in soils (Wilkinson et al., 1976), these pesticides are no-longer used in agriculture. Newer types of chemical insecticides used in the past two decades included pyrethroids, phenyl pyrazoles, and neonicotinoids (Jeschke et al., 2011), which in some cases function *via* pest repellency or morbidity, rather than mortality (van Herk et al., 2008; Vernon et al., 2009). However some of the neonicotinoids and phenyl pyrazoles are already prohibited due to negative effect on bees and other pollinators (Zhang and Nieh, 2015), aquatic invertebrates and fish (Werner and Moran, 2008) or beneficial spiders and mites (Douglas and Tooker, 2016).

Accordingly, several nonchemical methods for wireworm control were proposed including crop rotation (Willis et al., 2011), crop residue removal, biofumigation (Furlan et al., 2010), weeding (Schepl and Paffrath, 2007), trap and cover crop use (Rogge et al., 2017), mechanical soil disturbance and biological control (Reddy et al., 2014; la Forgia and Verheggen, 2019). Also entomopathogenic fungi (EPF) can significantly reduce insect pest pressures. Typically, they penetrate the insect cuticle, paralyse and destructively colonize insect bodies (Yousef et al., 2018). Several commonly occurring species of *Metarhizium* and *Beauveria* are known to be effective against wireworms (Kabaluk and Ericsson, 2007a; Kabaluk and Ericsson, 2007b; Kabaluk et al., 2013; Razinger et al., 2013; Reddy et al., 2014; Brandl et al., 2017; Rogge et al., 2017; Benjamin et al., 2018; Razinger et al., 2018b). They also infest wireworms as naturally occurring soil fungi (Kabaluk et al., 2005). In addition to causing pathogenicity in pest insects, EPF also have other beneficial functions as they enhance plant growth and mineral nutrition and exclude phytopathogens form rhizosphere niches (Herbst et al., 2017; Rivas-Franco et al., 2019; Ahmad et al., 2020).

Various studies have stressed that plants may actively shape their root microbial communities through rhizosphere depositions (Dennis et al., 2010; Canarini et al., 2019) and some have speculated that rhizosphere colonizing entomopathogenic fungi could protect plants from plant insect pests in tritrophic interactions (Vega et al., 2009; Bruck, 2010; Steinwender et al., 2015). Therefore, we hypothesized that utilizing rhizosphere competent EPF could contribute to potentially long-lasting pest management solutions, and decrease the amount of required biopesticide product. Accordingly, we are trying to identify microbial agents with plant beneficial metabolic or ecological traits that can be bioaugmented in the rhizosphere(Compant et al., 2010). In previous studies we screened the virulence of several EPF species (Razinger et al., 2013; Razinger et al., 2018b). The aim of this study was to apply EPF onto maize kernels as a one-step prophylactic strategy to protect maize plantlets during germination and sprouting against wireworm herbivory. In addition, the plant × microbe interactions were investigated in a series of laboratory experiments.

## Materials and Methods

### Wireworm Rearing

Wireworms of the species *Agriotes lineatus* L. were reared in a glasshouse following methods described by Kölliker et al. (2009). For collecting adult click beetles, a 5–10 cm thick layer of freshly mown grass was placed on top of a 2 m^2^ plastic (PVC) sheet placed from late April to late June on either meadows or grass areas juxtaposing agricultural fields. The grass on the sheet was inspected twice a week, and adults were collected. Beetles identified as *A. lineatus* were then placed in plastic containers with added food (dry baker’s yeast and honey mixture 1:9 w:w). After 24 h, 5–15 beetles were transferred to individual 10 L plastic pots filled with commercial planting soil (Potgrond H, Klasmann-Deilmann GmbH, Geeste, Germany). Wheat, barley, or maize was sown into the pots; small plastic cups filled with honey and yeast mixture were placed on top of the substratum to provide an additional food source for the adults. To prevent escape of insects, an insect rearing bag (BugDorm, Taiwan) was erected aboveground. Plants in pots were watered and wheat or corn seeds re-sown when needed. After approximately 10 months wireworms of average length 15 ± 1 mm were collected from the rearing pots. Rearing was performed in a glasshouse under natural illumination at 15–30°C and 40–65% RH.

### Fungi Collection and Growing

Entomopathogenic fungi such as *Metarhizium brunneum* (strains 1154, isolated from soil, and 1868, from dead *Agriotes* sp. adult), *M. robertsii* (1880, unknown host), and *Beauveria brongniartii* (from *Melolontha melolontha* L.), isolated from agriculturally used areas in Slovenia, were used for *in-vitro* mortality bioassays. The isolates are kept in the mycological collection of the Agricultural Institute of Slovenia. Fungal cultures were incubated at 24 ± 1°C in darkness for 14 d on full or 1/3^rd^ strength potato dextrose agar (PDA, Biolife Italiana S.r.l., Milan, Italy). Tween 80 (0.05%, Sigma-Aldrich, Germany) was used for preparing conidial suspensions (1 × 10^8^ spores ml^−1^) and conidial viability was assessed as in Razinger et al. (2014).

### Laboratory Experiments

#### Virulence and Pathogenicity Assessment

Wireworms were dipped for 10 s in 3.5 ml of spore suspensions with continuous gentle agitation. Additionally, Naturalis (Andermatt biocontrol AG, Grossdietwil, Switzerland, based on *Beauveria bassiana* ATCC 74040) (recommended concentration of 0.1 v/v) and Delfin (Andermatt biocontrol AG, Grossdietwil, Switzerland, based on *Bacillus thuringiensis* var. *kurstaki*) (0.05%, v/v) were used as reference biopesticides. Tween 80 (0.05%) was used as the negative control. Commercial potting substrate (Special substrate, Floragard, Germany) was steam treated for 1 h in loosely closed plastic bags, in which the top of the soil reached 92°C. Sterile 50 ml centrifuge tubes were filled with 25 ml of steamed substrate and moistened with 2.5 ml sterile demineralized water. One infected wireworm was placed on top of the substrate of each centrifuge tube and next to a 2 mm thick slice of organically produced carrot. The tubes were gently capped so that air could freely circulate. Ten wireworms were used per treatment and the experiment was repeated twice independently (n = 20). Experiments were observed on a weekly basis for 8 weeks. At each observation wireworms were classified as living, dead and dead and mycotic. Both experiments were carried out in an environmental chamber at 20 ± 1°C, 80 ± 5% relative humidity, in darkness.

#### Plant × Microbe Interactions

The laboratory experiments investigating plant × microbe interactions were performed in the absence of wireworms. Seeds were prepared according to Razinger et al. (2018a). Approximately 200 ml of wheat seeds of variety Renan and 1,200 ml of maize seeds of variety DKC4190 were surface disinfected by immersing seeds for 3 min in 70% ethanol with hand shaking, rinsing twice with sterile demineralized water and drying in a laminar flow chamber. For seed coating exposure, conidial suspensions of a concentration of 2 × 10^7^ viable conidia ml^−1^ were prepared in 1% carboxymethyl cellulose (CMC; Sigma-Aldrich Chemie GmbH, Steinheim, Germany). The amount of conidia attached to the seeds was estimated by washing the conidia off five seeds per fungal treatment with 0.05% Tween 80. The number of conidia was assessed by plating serial dilutions on 1.5% malt extract agar (Sigma-Aldrich Chemie GmbH, Steinheim, Germany). Washing and plating were performed in triplicate. The coating success was checked twice in triplicate (n = 6) and measured in means ± SE. Retrieved number of conidia were 7.0 × 10^4^ ± 6.1 × 10^3^ per maize seed and 9.4 × 10^4^ ± 3.9 × 10^4^ per wheat seed (*M. brunneum* 1154); 8.3 × 10^4^ ± 3.9 × 10^3^ (maize) and 6.6 × 10^4^ ± 2.5 × 10^4^ (wheat) (*M. brunneum* 1868); 1.2 × 10^5^ ± 1.3 × 10^4^ (maize) and 1.1 × 10^5^ ± 1.9 × 10^4^ (wheat) (*B. brongniartii* 1877); 1.0 × 10^5^ ± 1.7 × 10^4^ (maize) and 8.1 × 10^4^ ± 2.7 × 10^4^ (wheat) (*M. robertsii* 1880). No conidia were retrieved from the negative control, *i.e.*, untreated maize or wheat seeds, surface disinfected seeds, and surface disinfected seeds coated with 1% CMC without conidia. The fungal treatments were compared to the surface disinfected and CMC-treated controls. The CMC control was in turn compared to the untreated and surface disinfected control to determine if the CMC coating or surface disinfection had an effect on germination rate or plant growth.

The effect of fungal coatings or control treatments on the germination success was evaluated according to the International Seed Testing Association protocols. In brief, maize was sown into moist sand and incubated for 7 d at 20°C with 8:16 d:n regime, whereas wheat was placed on moist filter paper, chilled for 5 d at 7°C and then incubated for 7 d at 20°C with 16:8 d:n regime. In a second experiment, the effect of fungal coatings or control seed treatments on seedling emergence (evaluated on days 2, 3, 4, 7, and 15), plant biomass (fresh above- and belowground plant tissue weight and length), and rhizoplane and endophytic plant tissue colonization was assessed in quartz sand in a growth chamber 15 d post sowing (20°C, 16:8 d:n). Fungus-coated or non-coated seeds were sown 2 cm deep into moist sand. Both experiments were performed independently twice with four biological replicates containing 25 seeds each. The fresh above- and belowground plant tissue weight and length were measured on 16 plants per treatment per experiment repetition and rhizoplane and endophytic plant tissue colonization was assessed on three plants per treatment per experiment repetition.

The ability of strains to colonize rhizoplane and roots or leaves as endophytes was evaluated on plants from the second experiment 15 d after sowing according to Herbst et al. (2017) with slight modifications. Five 2-cm-long pieces of roots were sampled per plant to evaluate rhizoplane colonization. The root pieces were washed once with tap water and five times with sterile demineralized water. The washed root pieces were transferred to CTC agar medium (Fernandes et al., 2010), which promotes the growth of EPF semi-selectively. The plates were incubated for 14 d at 22 ± 1°C. Another collection of five washed root pieces and five washed leaf pieces (root pieces 2 cm in length; leaf pieces 1–2 cm^2^) per plant were surface disinfected for evaluating endophytic colonization by the fungi. Surface disinfection was performed in 50-ml centrifuge tubes by submersing pieces in 5 ml 70% ethanol for 3 min. During the 3 min of submersion in ethanol the tubes were vigorously vortexed three times for 10 s. Plant tissue pieces were then washed with sterile demineralized water. To evaluate the efficiency of the surface disinfection, 100 µl of the final wash-water was plated onto CTC plates. In all cases, no fungal colonies grew on the CTC plates from the final wash-water. Fungi emerging from washed or surface disinfected plant tissue pieces were isolated by transferring single hyphal tips to clean agar plates. After an incubation of 10–14 days at 25°C, retrieved fungal isolates were identified through morphological comparisons with strains used for inoculations.

### Field Experiments

#### Experimental Sites, Design and Crop Management

Four field experiments (three in 2017 and one in 2018) were conducted to assess the biopesticidal efficacy of *M. brunneum* strain 1868 against wireworms by comparing plants that emerged from 1868-coated and uncoated seeds. Three commonly used maize varieties (FeroXXY, LG 34.90 and Chapalu) were tested at two different locations in Eastern Slovenia. Location 1 was at Bučečovci (46°35′07″N, 16°06′37″E; 0.112 ha) on dystric planosol with FeroXXY sown on April 27, 2017 and Chapalu on May 9, 2018; location 2, Laporje (46°35′14″N, 15°61′04″ E; 0.112 ha) on dystric gleysol with FeroXXY and LG 34.90 sown on May 8, 2017. Both sites are characterized by mild continental climate, low-medium rainfall during maize growing season, no available irrigation, and a medium grain yield potential (<12 t ha^−1^). Two plots of the same size were designed for each maize variety. Each plot consisted of four maize rows spaced 0.7 m apart and of 100 m length giving 0.028 ha per plot. Approximately 2.400 seeds were sown per plot giving a theoretical plant stand of 85.000 ha^−1^. The same crop and weed management was applied for both plots in each experiment; thus the two plots differed only in the EPF seed treatment. All field experiments were managed with standard, farmer-owned equipment suited for field scale applications. The field experiments were evaluated in springtime to assess seedling emergence and wireworm damage to the seedlings. Pre-harvest-related parameters were assessed in autumn.

#### Wireworm Damage and Emergence Evaluation

Wireworm damage was evaluated 3 to 4 weeks after sowing at the 5- to 6-leaf stage. Four to six 20 m segments within the 100 m plots were randomly selected and marked with wooden poles. In these segments, all emerged plantlets were categorized into three groups: wireworm-damaged plantlets (*e.g.*, leaves exhibiting drilling holes, dead central leaf, yellow stripes on leaves), undamaged plantlets, and a sum of the previous two categories—total plantlet stand. The number of total spring observations per treatment (*i.e.* biological replicates, each consisting of >100 plants) was 10 for variety FeroXXY, four for LG 34.90 and six for Chapalu.

#### Pre Harvest Evaluation

In mid-September (2017) or the second half of August (2018) the previously marked segments were evaluated to assess the following parameters: final stand—reduced growth, no ears (*i.e.*, plants reduced in growth or damaged and having no ears), final stand—plants carrying corn ears, and final stand—total plant stand. Additionally, from each segment, 10 fresh plants (with ears) and ears with husks alone were weighed and the number of ears counted. The number of total autumn observations per treatment (*i.e.* biological replicates, each consisting of >100 plants) was 12 for variety FeroXXY, six for LG 34.90, and six for Chapalu.

### Statistical Evaluation of Data

Plant × microbe interaction data was first analyzed for normality of distribution by D’Agostino–Pearson omnibus K2 test. In case of normal distribution it was analyzed by one- or two-way analysis of variance, and in case significance was observed, individual treatments were subjected to Bonferroni’s multiple comparison *post-test*. When data was not normally distributed it was analyzed using the Kruskal–Wallis test followed by Dunn’s multiple comparison test (Motulsky, 1995). The time-based wireworm mortality was analyzed using Kaplan–Meier survival analysis. When multiple survival curves were compared, the significance threshold was corrected according to the Bonferroni method (Panevska et al., 2019). The field experiment data were analyzed by general linear model (GLM), where the effect of factor *treatment* (*M. brunneum* and negative control), *maize variety* (FeroXXY, LG 34.90 and Chapalu) and their interaction were analyzed on the dependent variables described in the section *Pre Harvest Evaluation*. Further, Fisher’s least significance difference (LSD) procedure at 95% confidence level was used to discriminate between the treatments within the field experiment dataset. The difference was considered significant at P < 0.05. If not stated otherwise, data presented are mean values ± standard error (SE). The number of biological replicates (n) is indicated in the figure or table captions. The analyses were performed with the statistical software Statgraphics Centurion XVI (StatPoint Technologies, Inc., The Plains, VA, USA) and GraphPad Prism 5.00 (GraphPad Software, Inc., La Jolla, CA, USA).

## Results

### Laboratory Experiments

#### Virulence and Pathogenicity Assessment

**Figure 1:**
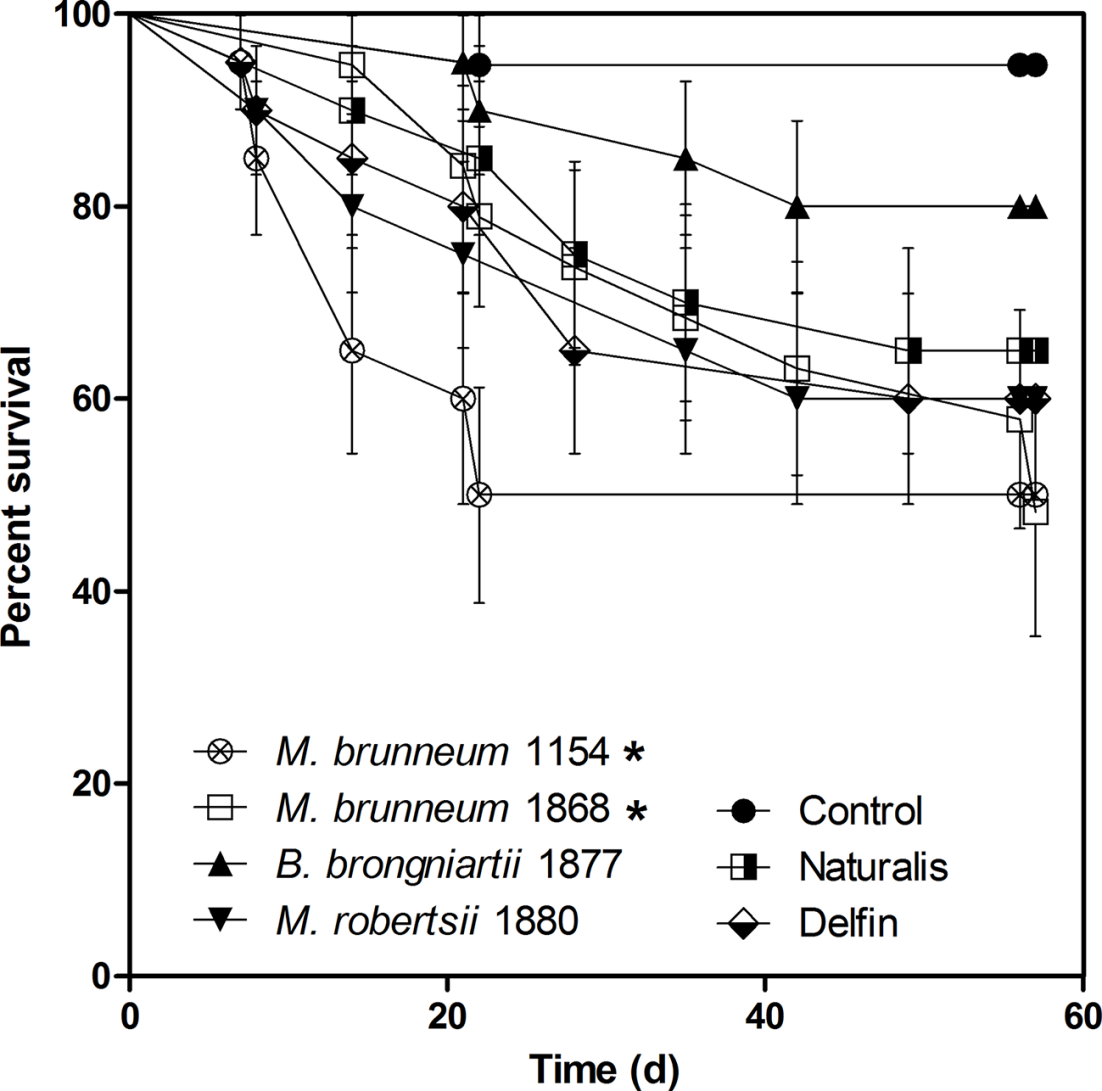
Mortality of wireworms treated with entomopathogenic fungi and two reference biopesticides in laboratory assays. The experiments were evaluated on a weekly basis for 8 weeks post infection. Asterisks denote significant difference from the control mortality (P < 0.05). Data from two independent experiments were pooled and analyzed with Kaplan–Meyer survival analyses (n = 20). Naturalis—commercial product based on *Beauveria bassiana*; Delfin—commercial product based on *Bacillus thuringiensis* var. *kurstaki*.

On average 5.6% of wireworms died in the negative control group for unknown reasons until day 56 of the laboratory experiments. Kaplan–Meyer survival analysis showed a significant mortality increase of wireworms treated with *M. brunneum* 1154 (50.0 ± 10.0% mortality) and 1868 (52.8 ± 2.78%). The other fungal strains and the Naturalis and Delfin biopesticide formulations did not cause a significant mortality increase during the 8-week experiment (**Figure 1**).

All fungal isolates and Naturalis formed mycelium and sometimes conidiogenous structures on wireworm surfaces. The highest incidence of sporulation was observed on wireworms treated with *M. robertsii* and Naturalis, where mycoses were observed on all dead insects. *Metarhizium brunneum* 1154 caused mycoses in 75% and strain 1868 on 80% of dead insects. *Beauveria brongniartii* sporulated on 67% of insect cadavers.

#### Plant × Microbe Interactions

##### Germination on Filter Paper

**Figure 2:**
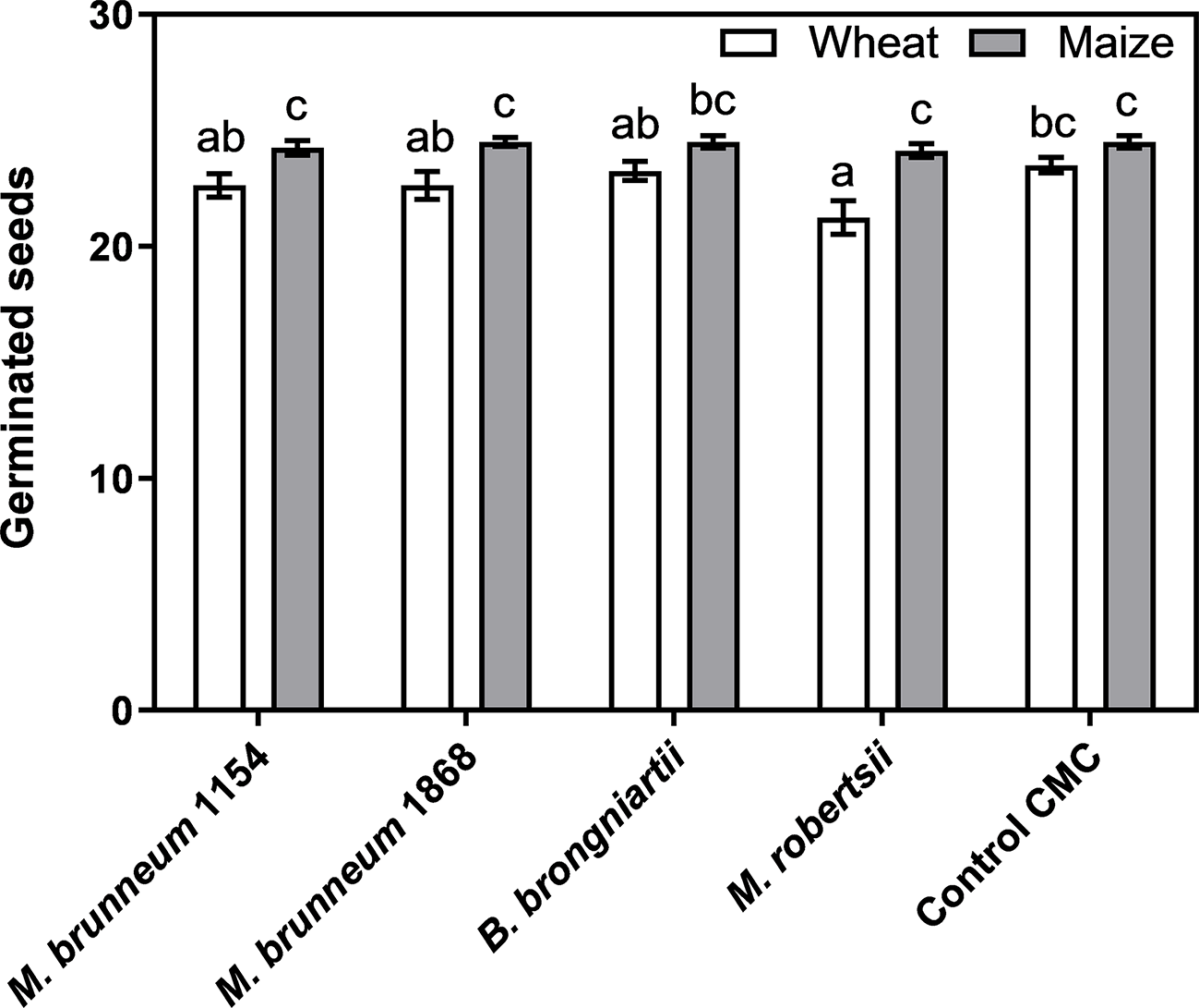
Maize and wheat seed germination after 7 d incubation on moist filter paper. Data from two independent experiments, each containing four biological replicates of 25 seeds each, were pooled and given as means ± standard error (n = 8). Bars not sharing the same lower-case letters are significantly different. *Control* *CMC*—seeds were surface disinfected and coated with 1% carboxymethyl cellulose.

Fungal treatment did not have any effect on maize seedling emergence when compared to the CMC control, while wheat seedling emergence was inhibited by *M. robertsii* (Kruskal–Wallis statistic and Dunn’s post-test, **Figure 2**). Seed disinfection or seed disinfection with CMC coating did not result in a significantly different seedling emergence when compared to untreated control seeds in both plant species evaluated (average germination of maize/wheat was 25 ± 0.3/24 ± 0.3, 24 ± 0.3/23 ± 0.7 or 24 ± 0.3/25 ± 0.3 for control CMC, disinfected seed or untreated seeds, respectively).

##### Speed of Seedling Emergence

A significant effect of factor treatment (F_6, 245_ = 14.0; P < 0.0001), time (F_4, 245_ = 1148; P < 0.0001) and their interaction (F_24, 245_ = 3.1; P < 0.0001) was observed on wheat germination timing. However, no differences were observed between CMC-treated surface disinfected control and fungal treatments. The only significant differences were observed between (i) CMC-treated, surface disinfected seeds and surface disinfected seeds at day 3 (P < 0.0001), and (ii) CMC-treated surface disinfected seeds and untreated seeds at days 2 (P < 0.01) and 3 (P < 0.0001). Consistently, seed germination was slower in CMC-treated and surface disinfected controls. No significant effects of seed treatment procedures were observed on the timing of maize seeds (not shown).

##### Effect on Plant Growth

**Figure 3:**
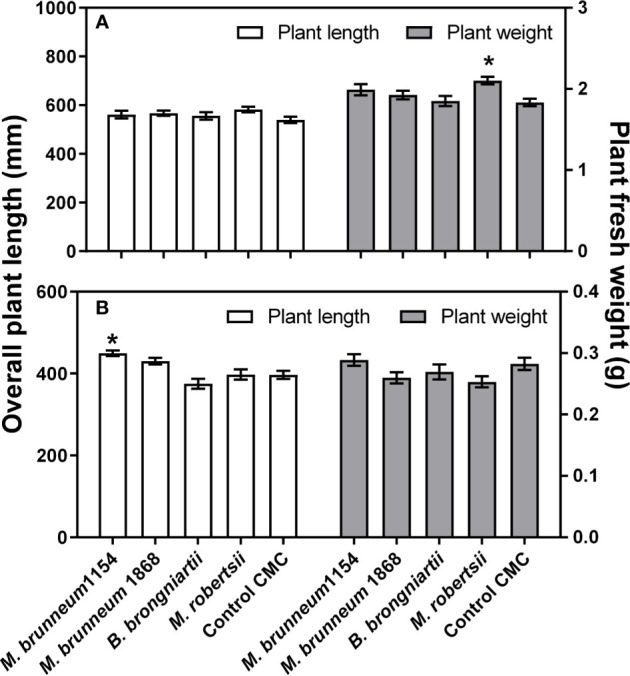
Maize **(A)** and wheat **(B)** plant length (left y-axis) and fresh weight (right y-axis). Thirty two plants from two independent experiments were analyzed. Data presented are means ± standard error (n = 32). Asterisks (*) denote significant difference from the respective negative controls (P < 0.05). *Control* *CMC*—seeds were surface disinfected and coated with 1% carboxymethyl cellulose.

Fungal treatments had a significant effect on plant fresh weight (P = 0.0054), but not on plant length (P = 0.2856) in maize, *e.g.* *Metarhizium robertsii* significantly increased the weight of maize plantlets compared to CMC-treated surface disinfected controls. In contrast, length of wheat plants (P < 0.0001) but not plant fresh weight (0.0857) was significantly affected by the fungal treatments in wheat, e.g. *M. brunneum* 1154 significantly increased wheat plantlets’ length compared to CMC-treated surface disinfected controls (**Figure 3**). Seed disinfection or seed disinfection with CMC coating did not result in a significantly different plantlet length or fresh weight compared to untreated control seeds in both crops (not shown).

##### Rhizoplane and Endophytic Plant Tissue Colonization

**Figure 4:**
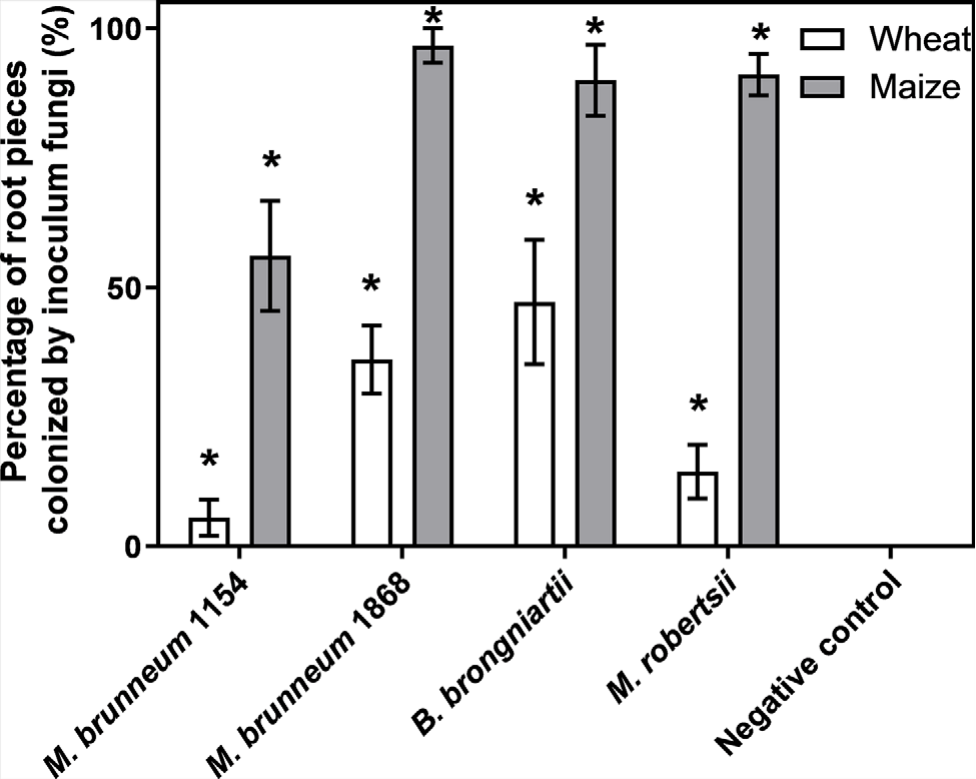
Frequency [%] of inoculated fungus re-isolation from rhizoplane of wheat or maize root pieces. Six plants from two independent experiments were analyzed. Data presented are means ± standard error (n = 6). Asterisks (*) denote significant difference from the respective negative controls (P < 0.05). The root pieces were vigorously washed prior to analysis.

Two-way ANOVA showed a significant effect of factors’ treatment (F_4, 49_ = 37.1; P < 0.0001) and plant species (F_1, 49_ = 124; P < 0.0001) and their interaction (F_4, 49_ = 9.22; P<0.0001) on rhizoplane colonization by the inoculated fungus. In all cases the inoculated fungi were significantly more often re-isolated from maize root pieces (**Figure 4**).

Endophytic tissue colonization was rarely observed. No endophytic colonization was observed in root or leaf tissue of wheat. Endophytic colonization of maize roots was observed for *M. brunneum* 1154 (16.7 ± 11.4%), *M. brunneum* 1868 (18.8 ± 9.3%), and *M. robertsii* (12.8 ± 6.6%). Endophytic colonization of maize leaves was observed only for *M. brunneum* 1868 (8.3 ± 5.7%).

### Field Experiments

The field experiments were evaluated in the springtime to assess seedling emergence and aboveground wireworm damage and in autumn, to assess harvest-related parameters. A significant effect of factor treatment and maize variety, but not their interaction, was observed on certain parameters assessed in the field experiments (**Table 1**). Treatment with *M. brunneum* resulted in a significant decrease of wireworm damaged plants and a significant increase of emerged undamaged plants during the spring evaluation in all three maize varieties tested. At harvest time a significant influence attributed to *M. brunneum* treatment was observed in the number of plants exhibiting reduced growth without ears, plants carrying corn ears and the number of total plants. However, in these three parameters also maize variety exhibited a significant effect. For example, only in the variety LG 34.90 a significant increase of the number of maize plants carrying corn ears was observed.

**Table 1:** Results of statistical analyses of the observed variables assessed in field experiments.

Maize variety	Chapalu		FeroXXY		LG 34.90		Significance		
Treatment	Control	*M. brunneum*	Control	*M. brunneum*	Control	*M. brunneum*	Maize Variety	Treatment	MV × T
Spring (emergence and wireworm damage) evaluation									
Wireworm damaged plants	12,17 ± 1,05^a^	3,17 ± 1,05^b^	11,50 ± 0,81^a^	3,10 ± 0,81^b^	9,50 ± 1,29^a^	3,75 ± 1,29^b^	ns	F_1, 39_ = 78,4; P = 0.0000	ns
Emergence—undamaged plants	90,50 ± 4,81^a^	106,50 ± 4,81^b^	95,40 ± 3,73^a^	108,30 ± 3,73^b^	86,50 ± 5,89^a^	102,25 ± 5,89^b^	ns	F_1, 39_ = 13,9; P = 0.0007	ns
Emergence—total plants	102,67 ± 4,64	109,67 ± 4,64	106,90 ± 3,60	111,40 ± 3,60	96,00 ± 5,69	106,00 ± 5,69	ns	ns	ns
Autumn (final stand) evaluation									
Final stand—reduced growth, no ears	9,33 ± 0,87^a^	8,67 ± 0,87^a^	3,25 ± 0,62^a^	2,83 ± 0,62^a^	4,33 ± 0,87^a^	0,83 ± 0,87^b^	F_2, 47_ = 37,9; P = 0.0000	F_1, 47_ = 5,55; P = 0.0232	ns
Final stand—plants carrying corn ears	80,00 ± 5,43^a^	86,17 ± 5,43^a^	100,03 ± 3,84^a^	102,82 ± 3,84^a^	86,50 ± 5,43^a^	105,33 ± 5,43^b^	F_2, 47_ = 7,63; P = 0.0015	F_1, 47_ = 5,25; P = 0.0271	ns
Final stand—total plants	89,33 ± 5,10^a^	94,83 ± 5,10^a^	103,25 ± 3,61^a^	105,67 ± 3,61^a^	90,83 ± 5,10^a^	106,17 ± 5,10^b^	F_2, 47_ = 4,02; P = 0.0253	F_1, 47_ = 4,15; P = 0.0479	ns
Fresh weight of 10 plants [kg]	5,47 ± 0,55	5,35 ± 0,55	6,64 ± 0,39	6,73 ± 0,39	9,84 ± 0,55	10,29 ± 0,55	F_2, 47_ = 39,9; P = 0.000	ns	ns
Weight of ears from 10 plants [kg]	2,67 ± 0,23	2,71 ± 0,23	2,71 ± 0,17	2,64 ± 0,17	3,77 ± 0,23	4,01 ± 0,23	F_2, 47_ = 20,1; P = 0.000	ns	ns
Number of ears on 10 plants	10,83 ± 0,69	10,67 ± 0,69	11,08 ± 0,49	10,92 ± 0,49	10,00 ± 0,69	10,33 ± 0,69	ns	ns	ns

Different lowercase letters indicate significant effect of fungal treatment within varieties. Ns, not significant; MV, maize variety; T, treatment.

**Figure 5:**
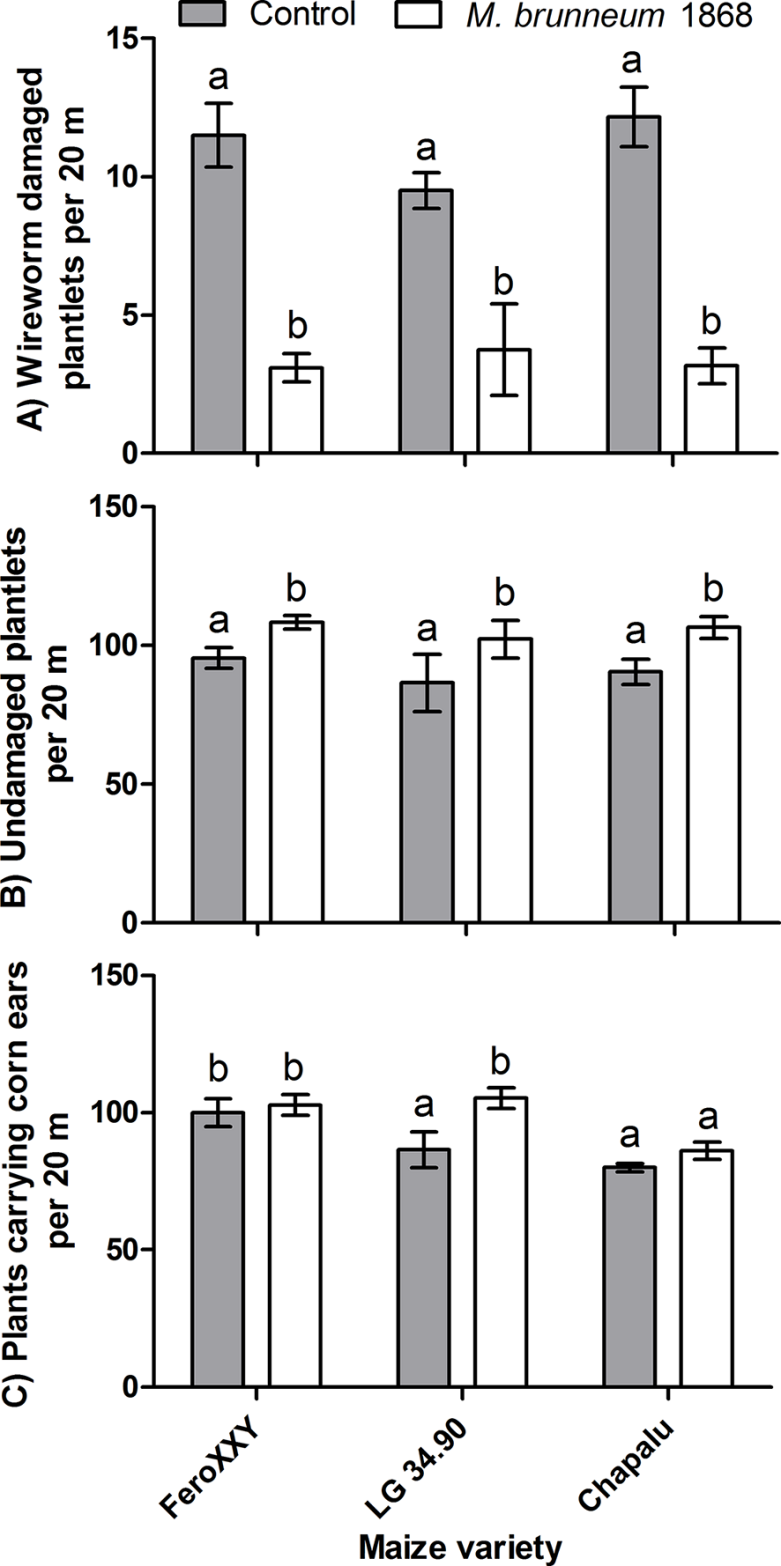
Field experiments: Effect of fungal treatment and maize variety on wireworm damage **(A)** and number of undamaged plantlets observed in spring **(B)** (n = 10 for variety FeroXXY, four for LG 34.90 and six for Chapalu), and number of plants carrying ears before harvest **(C)** (n = 12 for variety FeroXXY, six for LG 34.90 and six for Chapalu). Bars not sharing the same lowercase letter (a, b) are significantly different at P < 0.05.

Among other parameters observed at springtime, wireworm damage was significantly decreased and consequently the number of undamaged plantlets was significantly increased in all three maize varieties treated with *M. brunneum* 1868. However these beneficial effects were observed at harvest time only for variety LG 34.90, where the number of plants carrying corn ears was significantly increased (**Figure 5**).

The *M. brunneum* treatment resulted in a significant increase (+17.9%) in the number of plants carrying corn ears at harvest time in variety LG 34.90 (**Figure 5C**). This allowed us to calculate a theoretical fresh grain yield increase. Based on the measured weights of ears with husks and differences in numbers of plants carrying ears at harvest time, an increase of 5.23 t ha^−1^ fresh ear yield was calculated attributed to *M. brunneum* treatment for variety LG 34.90. Similarly, the *M. brunneum* treatment may reduce the wireworm related collapse of entire plants and could lead to an increase of 11.03 t ha^−1^ fresh aboveground biomass for variety LG 34.90. These calculations average fresh weight of 10 plants and fresh weight of ears from the same 10 plants and pooled data from treated and untreated maize variety LG 34.90 as the fungal treatment significantly affected only final stand of LG 34.90 plants carrying ears but not their fresh weight or the weight of fresh corn ears.

## Discussion

Of the several EPF strains tested in the laboratory, *M. brunneum* 1868 showed the highest virulence to *Agriotes lineatus* wireworms and was most often re-isolated from washed pieces of maize roots that emerged from seeds coated with conidia of that strain. Frequency of retrieved re-isolation events suggests that *M. brunneum* 1868 is rhizosphere competent. Based on these findings we evaluated its potential to reduce damages of wireworms in field settings. Maize plants stemming from kernels inoculated with *M. brunneum* 1868 conidia showed significantly less wireworm damage at emergence in the field experiment resulting in significantly higher undamaged maize stand in springtime. While the early plant belowground herbivory avoiding effect could be measured in all three varieties, increased fresh ear yield and fresh aboveground biomass was observed only in variety LG 34.90 at harvest time.

While chemical insecticides have an immediate effect on wireworm fitness, no such immediate effect can be expected through inoculating seeds or roots with entomopathogenic fungi (Razinger et al., 2013; Razinger et al., 2018b). Even after inoculating insect larvae directly with dense conidial suspensions, long incubation is required for observing an effect on insect larvae. In the laboratory experiments, wireworm mortality reached less than 60% after 8 weeks (**Figure 1**), whereas wireworms damage corn seedlings already within three to four weeks after sowing. It is therefore possible that the reduction of *Agriotes* herbivory by *M. brunneum* is due to larval repellence or other mechanisms. Also Kabaluk and Ericsson (2007a) reported that wireworms were not killed but repelled by *Metarhizium anisopliae* (Metchnikoff) Sorokin contaminated soil and that repellence increased with the conidial concentration in soil in laboratory experiments. Based on these results they postulated that the plant stand density in field settings increased possibly due to larval repulsion (Kabaluk and Ericsson, 2007b). Similar maize stand density increase was also observed when an encapsulated *M. brunneum* formulation, registered for wireworm management in potatoes (Brandl et al., 2017), was tested against wireworms in maize (prof. S. Vidal, personal communication). Metabolite production by endophytes of other species of the Clavicipitaceae or endophyte mediated production of volatile organic compounds has frequently been discussed as a mechanism resulting in herbivory repellence (reviewed in Johnson et al., 2016). Similarly, predatory bugs such as *Anthocoris nemorum* L. sense the presence of *Beauveria bassiana* after leaf inoculation with conidia of that species (Meyling and Pell, 2006). Meyling and Pell (2006) also observed that *A. nemorum* avoided contact with the thus inoculated leaves. It is thus possible that the high conidial inoculum mediated presence of *M. brunneum* can protect crop plants from wireworms non-parasitically.

*Metarhizium brunneum* did not stimulate early maize growth in the here described laboratory settings, which was also reported by Kabaluk and Ericsson (2007b) for *M.* *anisopliae*. Specifically, no effect of fungal treatment was observed on the speed of seed germination in maize and wheat. The only significant effect on the speed of seed germination was attributed to CMC-treatment used in the seed coating procedure, revealing a need for additional experiments and especially improved formulation for testing possible effects of *Metarhizium* on plant growth. By using a simple inoculation technique of immersing seeds in conidial suspension for 2 h, Ahmad et al. (2020) reported a *M. robertsii* mediated increase of maize height and aboveground biomass compared to control plants in laboratory experiments. Ahmad et al. (2020) also observed a much higher proportion of endophytically colonized maize leaves as we did, and they calculated a positive correlation between plant height and aboveground biomass and the proportion of endophytic root and leaf colonization by *M. robertsii*. *Metarhizium brunneum* strain 1868 well developed on maize root surfaces (**Figure 4**) and clearly better than on cauliflower (Razinger et al., 2014) or broccoli (Herbst et al., 2017) roots. This tight association with maize roots is in sync with the report by Hu and Bidochka (2020), who suggested that *Metarhizium* has a preference for monocots such as barley and corn. As a rhizosphere colonizer, *Metarhizium* spp. might also protect corn from other detrimental factors like soil pathogens (Kabaluk and Ericsson, 2007b; Vega et al., 2009). Furthermore, a higher frequency of endophytic colonization was detected in maize roots (18.8%) compared to leaves (8.3%). This could be the result of fungal preference towards different tissues within the plant host, *i.e.* plant root preference by *Metarhizium* species as postulated by Behie et al. (2015).

Laboratory results are often inconsistent with field trials (Kabaluk et al., 2005; Kölliker et al., 2011; Sufyan et al., 2017). To achieve the highest EPF mediated control effectiveness, many factors must be considered, such as landscape properties, soil characteristics, crop type, *etc.* In addition, different *Agriotes* species can be differently susceptible to a certain entomopathogenic taxon or individual strain or formulation product (Kölliker et al., 2011). The challenge is thus to find a strain or a mixture of different strains that performs equally well in different environmental conditions and against different pests. One might consider using multiple EPF strains of the same species to cover a wider range of ecological conditions, but such a strategy would be very difficult to put into practice due to registration constraints (Gadhave et al., 2016; Humber, 2016).

## Conclusions

The investigated entomopathogenic fungi exhibited multifaceted functions (Vega et al., 2009), *i.e.* pathogenicity to wireworms, rhizosphere competence and some growth promoting effects of maize and wheat plants in lab settings. However, observed effects were either depending on the EPF strain, plant species or variety. *Metarhizium brunneum* strain 1868 reduced *Agriotes* herbivory and increased initial plant stands of all three maize varieties tested in field settings. Interestingly, pre-harvest maize stand density and yield were increased only in one of the three varieties. Similar differential interactions between EPF and different plant varieties were reported by Canassa et al. (2020) for two root-inoculated strawberry varieties. This highlights the problems of generalizations and warrants further studies on the mechanisms of plant × fungus interactions. Insect repellence (Meyling and Pell, 2006; Kabaluk and Ericsson, 2007a) mediated through fungal rhizosphere competence may be the underlying mechanism for the measured increase of plant biomass. The ability of tested fungi to improve stand and robustness of young maize plants could contribute to wireworm stress resilience as this pest limits maize growth especially after crop emergence and less towards harvest time (Taupin, 2007).

## Data Availability Statement

All datasets generated for this study are included in the article/**Supplementary Material**.

## Author Contributions

JR provided the initial concept and design of the study and designed and led the execution of the field trial; JR and H-JS performed laboratory trials; JR and EP wrote the manuscript. H-JS contributed to study design and manuscript drafting. All authors contributed to the article and approved the submitted version.

## Funding

The research was financed partly by the Slovenian Research Agency (ARRS) (Agrobiodiversity program group, grant number P4-0072 and a grant to EP, 1000-18-0401), the Administration of the Republic of Slovenia for Food Safety, Veterinary Sector and Plant Protection (UVHVVR), Ministry of Agriculture, Forestry and Food (MKGP), the EU FP7 Project CropSustaIn (grant FP7-REGPOT-CT2012-316205), and H2020 projects EXCALIBUR (grant 817946) and ECOBREED (grant 771367).

## Disclaimer

This article reports the results of research only. Mention of trade names or commercial products in this publication is solely for the purpose of providing specific information and does not imply recommendation or endorsement by the Agricultural Institute of Slovenia.

## Conflict of Interest

The authors declare that the research was conducted in the absence of any commercial or financial relationships that could be construed as a potential conflict of interest.
